# Updates on Minimally Invasive Treatment of Adrenal Tumors

**DOI:** 10.3390/cancers18111728

**Published:** 2026-05-26

**Authors:** Dogukan Akkus, Eren Berber, Rafael Humberto Pérez-Soto

**Affiliations:** 1 Department of Endocrine Surgery, Cleveland Clinic, Cleveland, OH 44195, USA; akkusd@ccf.org (D.A.); berbere@ccf.org (E.B.); 2 Instituto Nacional de Ciencias Médicas y Nutrición, Salvador Zubirán, Mexico City 14080, Mexico

**Keywords:** adrenalectomy, robotic, minimally invasive, adrenal tumors

## Abstract

Adrenal tumors are increasingly diagnosed due to widespread use of cross-sectional imaging and an aging population, making adrenalectomy a progressively more common surgical procedure. Over the past three decades, adrenal surgery has undergone a paradigm shift from open adrenalectomy to minimally invasive (MI) techniques, with laparoscopic adrenalectomy becoming the standard approach for most benign and selected malignant adrenal tumors. More recently, retroperitoneoscopic and robotic approaches have expanded the armamentarium available to adrenal surgeons, allowing for tailored, patient-specific surgical strategies. This review summarizes current evidence on MI adrenalectomy techniques, including transperitoneal and retroperitoneal laparoscopic approaches, hand-assisted adrenalectomy, and robotic adrenalectomy, with particular emphasis on their role in pheochromocytoma and adrenocortical carcinoma. In addition, evolving ancillary technologies such as laparoscopic ultrasound, indocyanine green fluorescence imaging, artificial intelligence, and virtual and augmented reality are reviewed, highlighting their potential to enhance intraoperative decision-making, safety, and surgical precision. Current controversies, including the role of preoperative alpha-blockade, partial versus total adrenalectomy in hereditary pheochromocytoma, the oncologic adequacy of MI surgery for adrenocortical carcinoma, and the selective use of lymph node dissection, are discussed. Available evidence supports MI adrenalectomy as a safe and effective approach in carefully selected patients when performed by experienced surgeons in high-volume centers. Technological innovations continue to refine surgical planning, execution, and training, suggesting that the future of adrenal surgery will increasingly rely on precision-guided, personalized, and data-driven strategies. This review offers a timely and comprehensive synthesis of the evolving landscape of MI adrenalectomy, uniquely integrating current evidence across the full spectrum of surgical techniques with a critical appraisal of emerging ancillary technologies while addressing unresolved clinical controversies relevant to contemporary surgical practice.

## Simple Summary

Minimally invasive (MI) adrenalectomy is the gold standard for the treatment of malignant, indeterminate, and functional benign adrenal tumors. Since Gagner introduced the technique in 1992, many advances have been made to improve surgical outcomes, postoperative recovery, patient safety, surgical training, and ergonomics. Among these are single-port adrenalectomy, posterior or retroperitoneoscopic adrenalectomy, hand-assisted and robotic adrenalectomy, as well as new technology such as indocyanine green (ICG)-guided adrenalectomy, artificial intelligence (AI) for intraoperative imaging analysis, and virtual and augmented reality for intraoperative use. The present review includes an updated view of MI adrenalectomy.

## 1. Introduction

Adrenal tumors have an estimated prevalence of 1.4% in the general adult population, most of them being benign, non-functional tumors. This prevalence increases with age, from 0.2% in the 18 to 25-year age group to 3.2% in the population over 65 years [1]. Although this seems to be a rare condition, recent data have shown a 10-fold increase in the incidence rate of these tumors in the last three decades. This finding is explained mainly by an increase in the incidental discovery of small benign tumors (<4 cm) in the population aged 40 and older [2]. Given the increase in the age of the general population of developed countries, this condition is expected to become a more frequent clinical scenario in the upcoming years.

The surgical management of adrenal tumors has undergone a remarkable evolution over the past three decades, transitioning from open adrenalectomy to a diverse armamentarium of minimally invasive (MI) techniques tailored to individual patient and tumor characteristics [3]. The introduction of laparoscopic adrenalectomy (LA) represented a paradigm shift in adrenal surgery, establishing the MI approach as the preferred strategy for the majority of benign and selected malignant adrenal lesions, with subsequent studies consistently demonstrating reductions in postoperative pain, length of stay, and recovery time [4]. Subsequent refinements in surgical technique gave rise to the posterior retroperitoneoscopic adrenalectomy (PRA), which further reduced operative time, postoperative pain, and hospital stay by circumventing the peritoneal cavity and avoiding mobilization of surrounding viscera [5]. This approach is particularly advantageous for patients with bilateral adrenal disease, as it allows bilateral adrenalectomy without the need for patient repositioning [6]. More recently, robotic platforms have expanded the technical capabilities of the surgeons, with Piazza et al. and Hubens et al. reporting the first cases of robot-assisted adrenalectomy in 1999, and the subsequent adoption of the da Vinci system enabling three-dimensional visualization, enhanced instrument dexterity, and improved ergonomics [7,8]. This last trend in MI adrenalectomy is still in progress as more and more high-volume adrenal surgery centers adopt this technology [9,10].

Despite these advances, several important gaps persist in the current literature. The comparative evidence base for MI adrenalectomy techniques remains largely limited to retrospective, single-center studies with heterogeneous patient populations and short follow-up periods, precluding robust conclusions regarding long-term oncologic and functional outcomes. The oncologic adequacy of MI approaches for adrenocortical carcinoma (ACC) remains a subject of active controversy, with existing data insufficient to support definitive guideline recommendations. Furthermore, the clinical integration of emerging ancillary technologies, including indocyanine green (ICG) fluorescence imaging, artificial intelligence (AI), and augmented and virtual reality, has outpaced the generation of high-quality evidence, as prospective validation studies and standardized protocols for their use in adrenal surgery are largely lacking. Finally, unresolved clinical questions regarding preoperative alpha-blockade, the role of lymph node dissection, and the indications for partial versus total adrenalectomy in hereditary pheochromocytoma continue to challenge surgical decision-making in the absence of prospective randomized data.

In this context, the present review aims to provide a comprehensive and timely synthesis of the current evidence on MI adrenalectomy techniques with particular emphasis on the evolving role of ancillary intraoperative technologies and addresses key unresolved clinical controversies, with the ultimate goal of informing evidence-based surgical practice and identifying priorities for future research.

## 2. Methods

### 2.1. Literature Search Strategy

A systematic literature search was conducted across three electronic databases (PubMed, EMBASE, and Google Scholar) encompassing publications from January 2020 to May 2026. This date range was selected to achieve the most contemporary evidence; however, seminal studies published prior to 2020 were also incorporated where necessary to provide appropriate historical and clinical context.

Given the broad scope of this review, individualized search strategies were developed for each topic covered, combining Medical Subject Headings terms and free-text keywords relevant to MI adrenalectomy techniques, robotic adrenalectomy (RA), PRA, pheochromocytoma, ACC, indocyanine green (ICG) fluorescence imaging, AI, augmented reality (AR), preoperative alpha-blockade, lymph node dissection (LND), and partial adrenalectomy (PA) in hereditary pheochromocytoma. Search terms were adapted to the indexing conventions of each database.

### 2.2. Study Selection

Articles were considered eligible for inclusion if they met all of the following criteria: (a) study design consisting of an original research article, randomized controlled trial (RCT), or systematic review with or without meta-analysis; (b) human-based study population; and (c) full text available in the English language. Studies were excluded if they: (1) were deemed irrelevant to the topic by consensus of at least two of three reviewers; (2) full text was unavailable; (3) were classified as case reports or case series; or (4) had been formally retracted.

### 2.3. Screening and Data Extraction

Title and abstract screening, followed by full-text review of potentially eligible articles, was performed independently by two reviewers. Discordant decisions were resolved by a third reviewer through discussion and consensus. To minimize the risk of missing relevant publications, the reference lists of all included articles were manually searched, and any citations fulfilling the eligibility criteria were additionally incorporated.

### 2.4. Narrative Synthesis

Given the heterogeneity of study designs, patient populations, and outcome measures across the included literature, a formal quantitative meta-analysis was not performed. Instead, findings were synthesized narratively, with particular attention to study quality, level of evidence, and clinical applicability. Where evidence was limited or conflicting, this is explicitly acknowledged within the relevant sections of the review.

## 3. Results

### 3.1. Minimally Invasive Surgery

**Figure 1:**
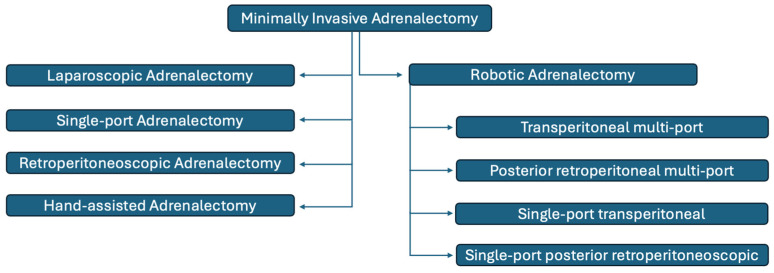
Schematic representation of the main minimally invasive adrenalectomy techniques.

A variety of minimally invasive surgical approaches to the adrenal gland are currently available. The selection of a technique is frequently guided by surgeon-specific factors, including training and experience, as well as institutional resources and individual practice patterns [11]. This section reviews recent advances in minimally invasive adrenal surgery, focusing on laparoscopic techniques such as laparo-endoscopic single-site adrenalectomy (LESSA), retroperitoneoscopic laparoscopic adrenalectomy (RPLA), and laparoscopic hand-assisted adrenalectomy (LHAA), as well as robotic adrenalectomy (RA). Figure 1 illustrates the categorization of minimally invasive adrenalectomy techniques.

#### 3.1.1. Laparoscopic Adrenalectomy

Since its initial description in 1992 as a transperitoneal procedure, LA has evolved to become the preferred surgical approach for the majority of adrenal tumors [4]. Lateral transabdominal adrenalectomy (LTA) is regarded as the gold-standard surgical approach for a broad range of functioning and non-functioning adrenal pathologies [12]. In addition, the widespread adoption of advanced surgical instrumentation over the last two decades, including ultrasonic, bipolar, and integrated radiofrequency energy devices, staplers, and hemostatic agents, has enabled reductions in operative time and an expansion of surgical indications with comparable safety rates [13]. Following the progressive accumulation of laparoscopic experience, a variety of novel instruments and operative techniques have been developed.

#### 3.1.2. Single-Port Adrenalectomy

The conventional multiport laparoscopic technique, which generally requires at least three ports to provide sufficient intraoperative exposure, is considered invasive [14]. LESSA offers excellent cosmetic outcomes with minimal visible scarring. Nevertheless, a steep learning curve and higher associated costs have restricted its widespread use, and its efficacy and safety remain controversial [15]. Several comparative studies have recently evaluated the outcomes of LESSA versus conventional laparoscopic adrenalectomy (CLA). A meta-analysis demonstrated significantly lower postoperative pain scores after LESSA than after CLA. However, subgroup analyses stratified by transperitoneal and retroperitoneal approaches revealed no significant differences in postoperative pain scores between the two techniques. Despite this, LESSA was associated with a significantly reduced relative risk of postoperative analgesic use. In addition, patients treated with LESSA experienced significantly shorter hospital stays than those undergoing CLA. In contrast, operative time was significantly longer for LESSA than for CLA [14]. Another meta-analysis demonstrated similar estimated blood loss, transfusion rates, conversion rates, and overall complication rates. However, operative time was significantly longer in the LESSA group. Postoperative pain was slightly lower in patients with LESSA than in those with CLA, and the length of hospital stay was shorter in the LESSA group. Cosmetic outcomes were comparable between LESSA and CLA [15]. A comprehensive longitudinal analysis comparing patient-reported satisfaction and cosmetic outcomes between subcostal (SC)-LESSA and trans-umbilical (TU)-LESSA and CLA demonstrated no significant differences in pain scores at any postoperative time point. In the CLA group, cosmesis and satisfaction scores were significantly lower at 3 months postoperatively. These scores gradually improved after 6 months, reaching levels comparable to those of the TU-LESSA group by 12 months postoperatively. No significant intergroup differences in cosmesis scores were observed among the three groups after 6 months. In contrast, satisfaction scores in the SC-LESSA group declined after 3 months and were significantly lower at 9 and 12 months postoperatively. Overall, female patients consistently reported greater concern regarding the surgical scar than male patients at all evaluated time points [16]. A specific concern in the LESSA literature is the inconsistency between the two cited meta-analyses regarding postoperative pain as the primary patient-reported outcome. This discordance is likely attributable to differences in included studies, operative approach (transperitoneal vs. retroperitoneal), and how pain was measured and reported across trials. The available evidence demonstrates that LESSA is a safe and feasible alternative to CLA. However, its implementation is dependent on the availability of specialized equipment and the surgeon’s level of experience. The technical challenges and limitations encountered with LESSA, particularly in confined operative fields, have prompted increased interest in alternative approaches that preserve minimal invasiveness while improving ergonomics and instrument control. In this context, retroperitoneoscopic adrenalectomy has emerged as a complementary strategy, as it provides direct access to the adrenal gland and avoids intraperitoneal manipulation. The single-port robotic system is particularly well suited for this approach, as the restricted retroperitoneal working space benefits from enhanced visualization and precise, wristed instrumentation, potentially overcoming several of the technical constraints associated with LESSA [17].

#### 3.1.3. Retroperitoneoscopic Adrenalectomy

RPLA has gained increasing popularity as an alternative surgical technique [18]. RPLA was first introduced by Mercan in 1994 and later established as a widely adopted technique through the work of Walz et al. in 1996 [19,20,21]. This growing adoption is largely attributable to several technical advantages of the retroperitoneal route, including avoidance of visceral mobilization, independence from prior abdominal surgeries, and the ability to perform bilateral adrenalectomy without patient repositioning [22]. Previous studies have suggested that RPLA can be technically challenging in patients with a high BMI or large adrenal tumors, primarily due to the limited available intraoperative working space [23]. Numerous studies comparing LTA and RPLA have demonstrated similar outcomes in terms of morbidity and mortality. However, the optimal surgical approach remains a matter of debate [18]. Two cohort studies reported that both transperitoneal and retroperitoneal approaches are safe, demonstrating comparable rates of major complications and mortality; however, the conversion rate was higher with LTA. Based on these findings, the authors suggested that RPLA may be best suited for smaller adrenal lesions [24,25]. In contrast, another cohort study found that RPLA is a favorable surgical technique, yielding patient outcomes comparable to those of LTA without compromising patient safety [18]. Three meta-analyses have demonstrated that RPLA is superior to LTA, offering reduced intraoperative blood loss and potentially shorter hospital stays [18,23,24,25,26]. In addition, RPLA appears to be associated with shorter operative time, decreased postoperative pain, and shorter hospitalization compared with LTA, while maintaining a comparable incidence of perioperative and postoperative complications, especially for adrenal masses smaller than 8 cm. Nevertheless, many surgeons continue to favor the standardized LTA approach, which consistently yields excellent outcomes, because transitioning to a reverse-angle anatomical approach poses a significant technical challenge [18]. Supporting this transition, a retrospective cohort study demonstrated that structured, intensive training programs significantly accelerated the learning curve for RPLA, resulting in reduced operative duration and shorter hospital stays. This study further showed that RPLA confers comparable or superior perioperative outcomes and is associated with significantly fewer and milder perioperative complications compared with LTA. Importantly, RPLA was also shown to be feasible and safe in patients with high body mass index and significant comorbidities [27].

Uniquely among the techniques in this section, RPLA has been evaluated in multiple RCTs, including the Barczyński et al. [28] and Chai et al. [29] trials, and a Cochrane review pooling five RCTs (*n* = 244) [30]. Despite this, the Cochrane review rated the overall certainty of evidence as very low to low using GRADE criteria, primarily due to small trial sizes, single-center designs, and risk of performance bias inherent to surgical trials. Notably, the conflicting findings between cohort studies, some favoring RPLA and others showing equivalence, are not fully resolved by the RCT data, which are themselves insufficiently powered to detect differences in rare complications. The learning curve study warrants specific caution; improvements attributed to structured training cannot be disentangled from the general effect of accumulated institutional experience over the same period. Despite the multiple benefits of RPLA, the technique can be associated with significant technical challenges, particularly in patients with large adrenal tumors. Integration of robotic platforms helps overcome these limitations by enhancing precision, ergonomics, and safety in complex retroperitoneal adrenal surgery [22].

#### 3.1.4. Hand-Assisted Adrenalectomy

LHAA was first reported in 2002 by Bennett and Ray [31]. The advantages of this technique include the benefits of MI surgery combined with the tactile feedback of the surgeon’s hand, which facilitates dissection, traction, and retraction of tissue planes, expedites dissection, and allows effective hemostatic compression in the event of bleeding. Furthermore, in the setting of malignant adrenal tumors, MI approaches raise additional concerns regarding capsular disruption and tumor spillage, with an associated risk of port-site seeding during specimen retrieval [32]. In this context, LHAA has emerged as a valuable alternative, as it preserves MI access while allowing direct manual control to enhance hemorrhage management, specimen handling, and oncologic safety in complex adrenal cases. One disadvantage of this approach is that patient repositioning may be required in cases of bilateral adrenal disease [33]. Recent studies have suggested that LHAA may offer advantages over CLA in the management of large pheochromocytomas [32,33]. The management of large pheochromocytomas using CLA is challenging due to the risk of organ injury from adhesions, substantial intraoperative bleeding, marked hemodynamic instability, and prolonged operative time [34]. A retrospective study including 36 patients with large pheochromocytomas demonstrated that LHAA was associated with shorter operative time, reduced length of hospital stays, and faster recovery of bowel function. Notably, LHAA resulted in a shorter operative duration, less manipulation of adjacent organs, particularly the gastrointestinal tract, and minimal postoperative organ dysfunction. These factors likely explain the earlier resumption of oral intake and shorter hospitalization observed in patients undergoing LHAA [32]. Another retrospective case series demonstrated that LHAA is a safe and reproducible technique, offering advantages in the resection of large and malignant adrenal tumors. The authors also noted that LHAA may be associated with a shorter learning curve, as it combines MI access with tactile feedback. The study supports the role of this approach in facilitating safe removal of a broad spectrum of adrenal tumors, including large lesions and those with malignant pathology, while preserving the benefits of MI surgery [33]. The central evidentiary concern for LHAA is not merely study design but patient comparability. Both cited studies enrolled patients with large pheochromocytomas, a notoriously high-risk, hemodynamically complex subgroup, making it methodologically inappropriate to extrapolate reported outcomes to the broader spectrum of adrenal pathology. Furthermore, the reported shorter operative time and faster bowel recovery in LHAA likely reflect the avoidance of extensive laparoscopic dissection in a selected group with bulky tumors and adhesions, rather than an intrinsic technique advantage. Prospective data comparing LHAA with CLA across equivalent tumor size categories are needed before any generalized superiority claim can be supported. Taken together, these findings support the feasibility and safety of LHAA for large adrenal tumors and suggest that it may be an appropriate alternative to CLA in selected high-risk patients.

#### 3.1.5. Robotic Adrenalectomy

The evolution of adrenalectomy reflects a sustained commitment to advancing surgical techniques that prioritize improved patient outcomes while minimizing procedural invasiveness. From the foundational era of open adrenal surgery to the transformative development of laparoscopic, retroperitoneoscopic, and robotic approaches, each advancement has played a pivotal role in refining adrenal surgical care. In 2001, the first fully robotic transabdominal lateral adrenalectomy was reported by Horgan and colleagues [35]. After the initial description, early experiences with robot-assisted laparoscopic adrenalectomy were reported primarily in small series, until Winter and colleagues published the first larger cohort of 30 patients in 2006 [36]. With the introduction of robotic technology [7,8], several limitations of laparoscopy became increasingly apparent, including unstable visualization, camera-related orientation challenges, constrained instrument mobility, surgeon fatigue, tremor, and the absence of three-dimensional vision [12]. Subsequently, the broad dissemination of robotic platforms led to the development and progressive standardization of RA [37]. Despite its ergonomic advantages and favorable learning curve, RA is limited by substantial initial costs and the absence of tactile feedback. Continued technological developments, such as advanced 3D visualization, fluorescence-guided imaging, and AI integration, are increasingly enhancing the precision and applicability of RA [38]. Multiple studies have reported advantages of RA compared with CLA, supporting its efficacy in real-world clinical practice [39]. Just recently, our randomized clinical trial of 54 patients (27 in each arm) comparing RA vs. CLA found no differences in primary outcomes in terms of operative time, complication rates, length of stay, pain scores, and overall costs. Nevertheless, secondary outcomes showed a benefit of RA in surgeon ergonomics, lower workload (NASA-LTX scores), reduced blood loss, and fewer intraoperative technical interruptions (less camera cleaning). In addition, no conversion to open surgery was required in the RA groups versus 15% in the CLA group. Collectively, these findings highlight that, while perioperative outcomes are comparable, RA offers meaningful advantages in operative stability and surgeon performance, potentially enhancing both surgical efficiency and safety [40]. Regarding the broader RA literature, claims of specific subgroup advantages in obesity, right-sided tumors, or bilateral disease are derived entirely from retrospective or observational data and should be explicitly labeled as hypothesis-generating. The evidence for robotic partial adrenalectomy in particular remains nascent, with no RCT data and outcomes largely confined to highly selected cases at expert centers.

Each RA approach offers distinct advantages and limitations. Therefore, careful consideration of patient-specific factors, including tumor size, anatomical location, and prior surgical history, is essential to selecting the most appropriate technique. Ongoing advancements in robotic technology have led to the development of multiple adrenalectomy approaches tailored to specific disease characteristics and operative challenges. These techniques can be broadly categorized into four main types, each with unique technical and clinical implications:

- Transperitoneal multi-port RA;
- Posterior retroperitoneal multi-port RA;
- Single-port transperitoneal RA;
- Single-port posterior retroperitoneoscopic RA.

Patient selection remains critical for optimizing RA outcomes. Tumor size, location, functionality, and laterality all influence intraoperative complexity. For example, right-sided adrenal tumors often present greater technical challenges due to their proximity to the inferior vena cava. This situation may be better managed with the enhanced dexterity, precision, and vascular control afforded by robotic systems. Similarly, large tumors, hormonally active lesions, prior abdominal surgery, and bilateral disease may further increase procedural complexity and should be carefully considered when selecting the most appropriate robotic approach [38]. Obese patients, who often present with increased intra-abdominal fat and a reduced working space, may particularly benefit from RA, as robotic instrumentation provides enhanced reach, articulation, and stability within restricted operative fields [41]. Furthermore, in the era of MI surgery, PA has emerged as a feasible surgical option for the management of small (3–4 cm), well-circumscribed adrenal lesions. This approach is particularly relevant in patients with bilateral disease; in hereditary syndromes such as multiple endocrine neoplasia type 2, von Hippel–Lindau syndrome, and familial pheochromocytoma; and in selected patients with unilateral, benign, hormonally active tumors, including aldosterone- or cortisol-producing adenomas. Preservation of endogenous adrenal function reduces postoperative steroid dependence and has been associated with favorable perioperative outcomes, including reduced blood loss, shorter operative time, and lower overall complication rates. Despite these advantages, the broader adoption of PA remains limited by technical complexity and the requirement for advanced surgical expertise. In this context, robotic platforms may mitigate some of these technical challenges compared with conventional laparoscopy by providing enhanced visualization and instrument dexterity [42], potentially facilitating precise cortical-sparing resection and preservation of venous drainage when technically feasible [43].

Despite continued advancements in robotic instrumentation, the absence of tactile feedback remains a recognized limitation. To address this constraint, the robotic surgery community has actively explored developing force-feedback systems. Although full haptic integration is still under investigation, emerging sensor technologies and experimental platforms suggest a future in which robotic surgery may incorporate reliable, real-time force perception [44]. AR and surgical navigation systems are also being introduced into RA, with early studies demonstrating the feasibility and potential utility of integrating preoperative CT and MRI datasets into the operative field to enhance anatomical orientation and intraoperative decision-making [45]. These technologies enable intraoperative guidance, support the achievement of safe resection margins, and help minimize injury to adjacent critical structures. In parallel, AI and machine learning (ML) are gaining increasing attention within the robotic surgery literature. Emerging applications include predictive analytics for surgical planning, AI-based image segmentation for accurate tumor localization, and real-time intraoperative decision support. In addition, ML algorithms are being incorporated into surgical training simulators to objectively assess performance, provide feedback, and facilitate skill acquisition [38]. The incorporation of ML algorithms holds significant potential for predicting patient-specific risks, refining preoperative planning, and supporting real-time intraoperative decision-making, thereby enhancing procedural safety and efficiency. In parallel, personalized medicine approaches, enabled by the integration of genomic, imaging, and biochemical data, may support tailored surgical strategies based on individual patient profiles, with the potential to improve oncologic and functional outcomes while reducing postoperative complications [17]. The discussion of emerging technologies (haptic feedback, AR navigation, AI/ML integration) in RA is based predominantly on early feasibility studies, small proof-of-concept series, and expert opinion, representing the lowest levels of clinical evidence. While these innovations are promising, claims regarding their clinical utility and impact on patient outcomes remain speculative in the absence of controlled studies with defined outcome endpoints.

#### 3.1.6. Overall Limitations of the Evidence Base for Minimally Invasive Adrenal Surgery

Several overarching methodological limitations characterize the available evidence across all MI adrenal surgery techniques reviewed in this section. The vast majority of comparative data derive from retrospective cohort studies, case series, and meta-analyses of predominantly non-randomized studies, all of which are inherently subject to selection bias. In most series, surgeons selected specific techniques based on tumor characteristics, patient anatomy, and institutional preference, introducing systematic confounding that cannot be fully adjusted for in retrospective analyses. As a result, reported differences in operative time, blood loss, hospital stay, and complication rates between techniques may reflect case-mix differences as much as true technical superiority.

Substantial clinical heterogeneity further limits the synthesis of evidence across studies. Included patient populations vary considerably with respect to tumor size, pathology (functioning vs. non-functioning, benign vs. malignant), BMI, comorbidity burden, and prior surgical history. Outcome definitions, including criteria for conversion, major complications, and postoperative pain assessment, are inconsistently applied across studies, complicating direct inter-study comparisons. These sources of heterogeneity are particularly pronounced in the meta-analyses cited throughout this section and should be considered when interpreting pooled estimates.

The availability of RCT evidence varies considerably across MI adrenal surgery techniques. For RPLA versus LTA, the strongest RCT evidence exists, and multiple completed trials are available, including the landmark Barczyński et al. trial [28] and the Chai et al. trial [29], with a review pooling five RCTs totaling 244 patients [30]. For RA versus CLA, RCT data also exist, including a trial specifically in pheochromocytoma patients (*n* = 140) [46] and our more recent trial (*n* = 54) [37], though both are limited in sample size and generalizability. For LESSA, a single RCT has been identified in the literature and incorporated within one meta-analysis [14], but the evidence base remains sparse, and further adequately powered trials are still explicitly called for. For LHAA, no RCT data are currently available, and the evidence rests entirely on retrospective series.

Taken together, the RCT evidence base for MI adrenal surgery is heterogeneous across techniques: relatively more developed for RPLA and emerging for RA but still limited for LESSA and absent for LHAA. Even where RCTs exist, sample sizes are generally modest and rarely powered to detect differences in low frequency but clinically important outcomes such as major vascular complications or long-term oncologic recurrence. The conclusions drawn throughout this section should therefore be interpreted with appropriate caution, and adequately powered, prospective comparative studies with standardized outcome definitions are needed to establish definitive evidence hierarchies across MI adrenal surgery approaches (Table 1).

### 3.2. Adrenalectomy for Pheochromocytoma

#### 3.2.1. Preoperative Alpha-Blockade

Recent evidence has challenged the dogmatic use of preoperative alpha-adrenergic blockade for patients with pheochromocytoma to reduce intraoperative hemodynamic instability and cardiovascular complications. Schimmack et al. published a meta-analysis on the role of alpha-blockade versus no blockade in 603 patients undergoing adrenalectomy for pheochromocytoma. No significant differences in intraoperative systolic (0.18 mmHg [−9.47, 9.83], *p* = 0.97) or diastolic blood pressure (1.45 mmHg [−2.92, 5.81], *p* = 0.52), heart rate (−2.21 bpm [−10.35, 5.93], *p* = 0.59), cardiovascular complications, or mortality between groups were found [48]. Unfortunately, the included studies were mostly retrospective and heterogeneous, making these findings based on low-quality evidence. In the same controversy, Wang et al. published their 2023 meta-analysis on the use of alpha-blockade in surgical patients with pheochromocytoma/paraganglioma, showing no benefit of alpha-blockade for intraoperative hemodynamic parameters (including hypertensive or hypotensive episodes), even in biochemically silent tumors. It is important to mention that a higher risk of prolonged postoperative hypotension requiring vasopressors was found in the alpha-blockade group (RR 4.21 [1.17, 15.18], *p* = 0.03) [47].

To increase the complexity of this topic, Zawadzka et al. analyze the use of selective versus non-selective alpha-blockade drugs in patients with pheochromocytoma undergoing surgery. This meta-analysis showed better intraoperative blood pressure control but higher use of intraoperative vasodilators with selective drugs (OR 2.46 [1.44–4.20], *p* = 0.001), without affecting overall morbidity or mortality. Interesting, selective alpha-blockade was associated with a borderline shorter length of stay (MD −0.58 days [−1.12, −0.04], *p* = 0.04) [49]. This contrasts with the findings of Buitenwerf et al. in their randomized controlled trial comparing the use of phenoxybenzamine and doxazosin, in which the authors found better intraoperative hemodynamic stability with phenoxybenzamine in terms of the number of events with systolic blood pressure >160 mmHg and duration of these episodes [50].

Current literature on these topics indicates a paradigm shift in adrenal surgery, moving from routine preoperative alpha-blockade to more individualized perioperative management. This remains under debate, and the omission of routine alpha-blockade should be restricted to highly selective cases in experienced multidisciplinary teams.

#### 3.2.2. Partial/Cortical Sparing Adrenalectomy

Current debate exists regarding total adrenalectomy versus partial or cortical-sparing adrenalectomy for patients with hereditary or bilateral pheochromocytoma. Total adrenalectomy improves outcomes for local recurrence but carries the risk of permanent adrenal insufficiency, whereas PA preserves cortical function at the cost of a higher risk of local recurrence.

In the past decade, Zawadzka et al. published a systematic review and meta-analysis on this topic, including a total of 25 observational studies (*n* = 1444) on patients with bilateral pheochromocytoma. Pooled analysis showed reduced risk for steroid dependency and acute adrenal crisis in the PA group (RR = 0.32, 95% CI [0.26–0.38] and OR 0.20, 95% CI [0.10–0.91], respectively). However, tumor recurrence risk was higher for this group compared with total adrenalectomy (OR 3.72, 95% CI 1.54–8.96), with no implications on metastasis or overall mortality rates [51]. Similar conclusions were reported in a meta-analysis by Schiavone et al., including 1202 patients with bilateral pheochromocytomas from 10 retrospective studies. Results showed a higher rate of recurrences (14.1 vs. 2.6%; OR 4.91, 95% CI [1.30–18.54]) and lower steroid dependence (11.6% vs. 93.3%; OR 0.003, 95% CI [0.0003–0.03]) for the PA group [52]. Complementary to these studies, in 2025, a systematic review and meta-analysis by Araujo-Castro et al. found biochemical and clinical cure rates of 99.7% and 99.8%, respectively, with a pooled complication rate of 5.9% for the PA in patients with hereditary/bilateral pheochromocytomas [53].

Finally, it is noteworthy to mention that even after PA, bilateral interventions may still result in a clinically relevant burden of adrenal insufficiency, around 65%, as described by Procopio et al. in their study on PA for MEN2-associated pheochromocytomas [54].

Taking this evidence all together, PA offers a clear advantage in reducing long-term steroid dependency and adrenal crisis risk with inferior local oncologic outcomes compared with total adrenalectomy. Decision-making should be individualized, weighing the hereditary risk profile, expected bilaterality, feasibility of follow-up, and the patient’s tolerance for lifelong steroid replacement versus monitoring for recurrence.

#### 3.2.3. Laparoscopic Versus Robotic Adrenalectomy

Since the worldwide adoption of robotic platforms, RA has gained increasing acceptance, particularly in high-volume centers. Several systematic reviews and meta-analyses comparing laparoscopic and robotic approaches to adrenalectomy have consistently shown comparable safety profiles, with no differences in overall complication rates, length of stay, readmissions, or transfusion requirements. Furthermore, conversion to open adrenalectomy appears to be less frequent in robotic-assisted procedures, suggesting a potential benefit in technically challenging cases [55,56].

For patients with pheochromocytoma, the evidence becomes more nuanced. For instance, a recent randomized controlled clinical trial demonstrated shorter operative time (92.5 vs. 122.5 min, *p* = 0.007) and reduced blood loss for the RA group of patients with highly secreting pheochromocytomas at the expense of significantly higher cost [46]. These findings have been supported by other recent published meta-analyses focusing specifically on pheochromocytoma patients, confirming the benefits of RA in terms of blood loss and length of stay, with no differences in operative time, conversion rates, and overall complications [57,58,59,60]. Interestingly, some of these studies have suggested a role for the robotic approach in intraoperative hemodynamic stability, particularly in large and highly vascularized pheochromocytomas, which might be associated with tremor filtration and the gentle tissue handling characteristic of robotic platforms [58,60].

Current evidence suggests that RA offers a modest perioperative advantage over laparoscopy for these tumors; however, the higher economic burden and longer learning curve associated with robotic platforms remain important limitations to its widespread adoption [57,60].

#### 3.2.4. Retroperitoneoscopic Versus Transabdominal MI Adrenalectomy

In 2020, Jiang et al. published a meta-analysis comparing retroperitoneoscopic and transabdominal MI adrenalectomy in patients with pheochromocytoma. Their study showed significantly shorter operative time (WMD: 34.9, 95% CI [27.02, 42.80] min, *p* < 0.01), reduced blood loss (WMD: 139.3, 95% CI [125.3, 153.2] mL, *p* < 0.01), and shorter length of stay (WMD: 2, 95% CI [1.18, 2.82] mL, *p* < 0.01) with the retroperitoneoscopic approach, with no effect on overall complication rates, conversion to open surgery, or intraoperative hemodynamic crisis [61]. More recently, Zhang et al. published a larger systematic review and meta-analysis on this topic, with nearly 600 patients, confirming the results of Jiang et al.’s study. However, the latter group noted a slightly higher rate of intraoperative hemodynamic instability in the retroperitoneoscopic group (OR 1.54, 95% CI [1.03, 2.29], *p* = 0.03), highlighting the importance of experienced anesthetic management and careful patient selection for this approach [62].

The evidence points towards a safe profile and better intraoperative outcomes for the retroperitoneoscopic approach in patients with pheochromocytoma.

### 3.3. Adrenalectomy for Adrenocortical Carcinoma

Complete surgical resection with negative margins (R0 resection) remains the cornerstone of curative treatment for adrenocortical carcinoma (ACC), which is strongly associated with improved recurrence-free and overall survival. Since tumor spillage, capsular disruption, and peritoneal dissemination are the main concerns during MI adrenalectomy, open adrenalectomy is still considered the gold standard treatment for ACC patients [63].

Recent meta-analyses comparing MI with open adrenalectomy (OA) showed the known benefits of MI surgery over open approaches in terms of reduced blood loss (WMD −1761.96, *p* = 0.016), shorter length of stay (WMD −2.96, *p* = 0.048), and faster postoperative recovery. These benefits may come at the cost of higher rates of positive surgical margins (RR 1.56, *p* = 0.018) and earlier recurrence (WMD −8.42, *p* = 0.048), especially in large or locally invasive tumors [64]. Nevertheless, careful patient selection for MI adrenalectomy—particularly for ENSAT stage I-III tumors or small adrenal tumors without radiologic evidence of local invasion—offers a risk-benefit profile potentially superior or at least equivalent to OA [64,65].

RA offers enhanced dexterity, three-dimensional visualization, and improved ergonomics when compared with LA, allowing for precise dissection, especially in anatomically challenging cases or very obese patients. However, current evidence has not demonstrated clear oncologic benefits of RA over LA in ACC patients, and its use should be restricted to selected cases in high-volume centers with high-volume surgeons [64,65].

#### Role of Lymph Node Dissection

Although lymph node involvement is associated with a worse prognosis in ACC, LND remains inconsistently performed during adrenalectomy. Recent observational studies and meta-analyses suggest that LND may improve disease-specific survival among patients with localized disease (ENSAT stage I–III) [66,67]. In contrast, no benefit of LND has been demonstrated for locally advanced or metastatic disease in ACC, and there is a gap in the current medical literature regarding a therapeutic advantage of LND supported by prospective studies [66,67]. In the pediatric population, retroperitoneal LND does not clearly improve oncological outcomes in completely resected tumors, highlighting the biological and clinical heterogeneity across age groups [68].

In summary, current evidence supports a more selective, rather than routine, use of LND for patients with localized ACC.

### 3.4. Ancillary Tools

#### 3.4.1. Laparoscopic Ultrasound Imaging

Laparoscopic ultrasound (LUS) emerged as a valuable tool for adrenal gland identification, tumor localization, and adrenal dissection in a complex retroperitoneal environment, compensating for the limited tactile feedback during MI adrenalectomy. Updated evidence on this tool is limited, likely due to the appearance of new technologies offering similar benefits, such as ICG fluorescence. In 2020, Sebastian et al., and in 2025, Mihai et al., showed the benefits of LUS in MI adrenalectomy through objective perioperative outcomes. Both studies provide evidence of shorter operative time, reduced blood loss, lower conversion rates, and faster patient recovery (earlier return to oral intake and reduced postoperative analgesic needs) when LUS is used for decision-making during MI adrenalectomy compared with standard MI adrenalectomy [69,70]. Additional advantages of LUS are that it does not require disposable consumables or patient-specific supplies. Once the ultrasound platform and laparoscopic probe are acquired, the technology can be used across multiple procedures and patients, representing a single capital investment. Furthermore, no contrast media or dyes are needed, thereby avoiding the risk of allergic reactions.

#### 3.4.2. Indocyanine Green Imaging

**Figure 2:**
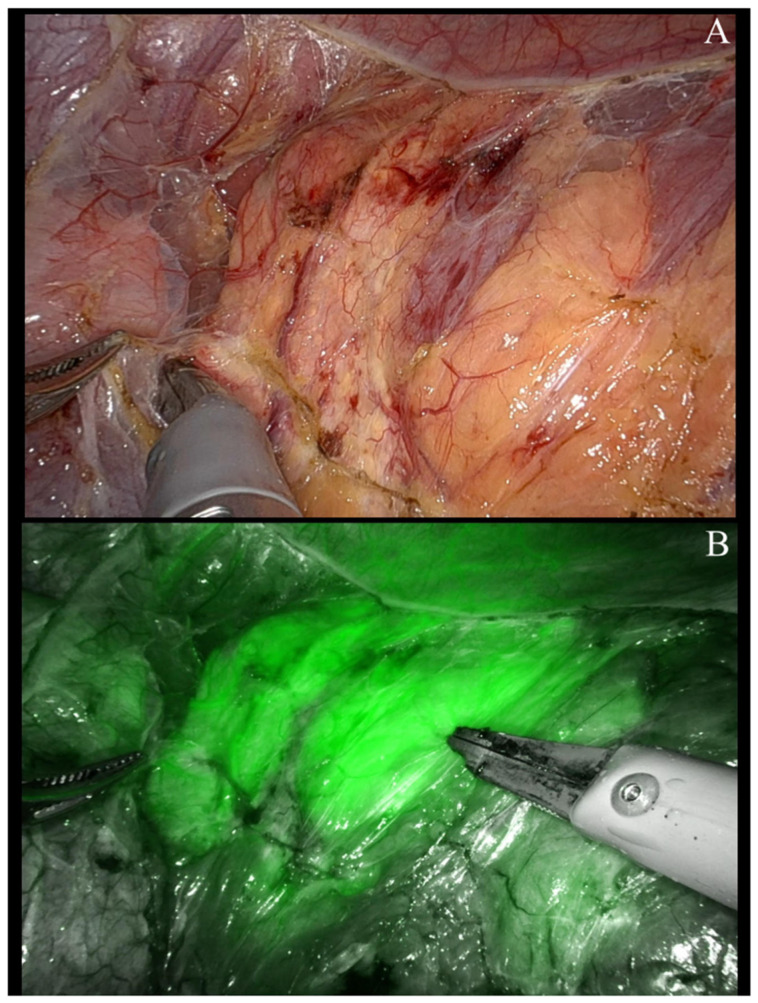
RBG (Red, Blue, and Green) vision of the left adrenal gland during robotic adrenalectomy (**A**), indocyanine green fluorescence view of the same case (**B**).

ICG fluorescence imaging has evolved as a valuable tool in MI adrenalectomy, exploiting the high vascularity of adrenal tissue to enhance its intraoperative visualization. ICG use during MI adrenalectomy enables rapid identification of the gland, improves discrimination from the surrounding retroperitoneal fat, and allows early intraoperative recognition of vascular structures (Figure 2).

Surgical robotic and laparoscopic cohorts have reported improved subjective adrenal tissue distinction and easier adrenal anatomy identification using ICG fluorescence, without associated adverse events, remarking on the safety and feasibility of its use during MI adrenalectomy [71,72]. Quantitative analyses further demonstrated that ICG fluorescence provides a wider contrast gradient between adrenal tissue and retroperitoneal fat than conventional imaging, supporting its perceived intraoperative benefit [73].

A major area of interest has been the use of ICG fluorescence patterns to distinguish between different adrenal tumors. Multiple studies have shown that adrenocortical tumors are typically hyperfluorescent, in contrast to medullary tumors such as pheochromocytomas, which often appear hypo- or isofluorescent relative to normal adrenal cortex tissue. In the largest published series, nearly 50% of pheochromocytomas demonstrated fluorescence, with a loss of fluorescence when tumors are larger than 3.2 cm [72]. This highlights the effect of tumor size on the fluorescence pattern of pheochromocytomas.

Another potential role for ICG is during cortical-sparing or partial adrenalectomy. Seeliger et al. in 2020 described the ability of ICG fluorescence to distinguish perfused from ischemic adrenal tissues, especially during retroperitoneoscopic adrenalectomy [74].

Furthermore, a recent prospective study evaluation of ICG-guided partial MI adrenalectomy showed that intraoperative ICG use altered the planned surgical strategy in over 70% of the cases, leading surgeons to move from partial to total adrenalectomy when fluorescence suggested inadequate remnant volume, poor vascularization, or isoflurescent tumors indistinguishable from normal cortex [75]. Interestingly, cases that fulfilled optimal criteria for partial adrenalectomy remained with appropriate cortical function with disease-free resection margins.

Beyond improving anatomical orientation and tissue discrimination, accumulating data suggest a growing role for ICG in intraoperative risk stratification and real-time decision-making, particularly in partial adrenalectomy and complex MI adrenalectomy.

#### 3.4.3. Artificial Intelligence

AI applications in adrenal surgery have focused mainly on radiomics and preoperative image segmentation to improve anatomical characterization of vascular and tumor-normal tissues after 3D imaging construction. Recent literature on intraoperative imaging guidance for MI adrenalectomy over the last decade is scarce. In 2023, Sengun et al. published the first study of AI for intraoperative imaging analysis during MI adrenalectomy. In the study, the authors developed a deep learning model to accurately identify the location of the left adrenal vein, guiding less experienced surgeons [76]. The same authors published, in 2024, an adaptation of the same model for MI right adrenalectomy, demonstrating the model’s high accuracy in identifying anatomical landmarks in real time, thereby improving safety during adrenal venous control [77].

AI applications for intraoperative imaging recognition during MI adrenalectomy are still an area under development.

#### 3.4.4. Virtual and Augmented Reality

**Figure 3:**
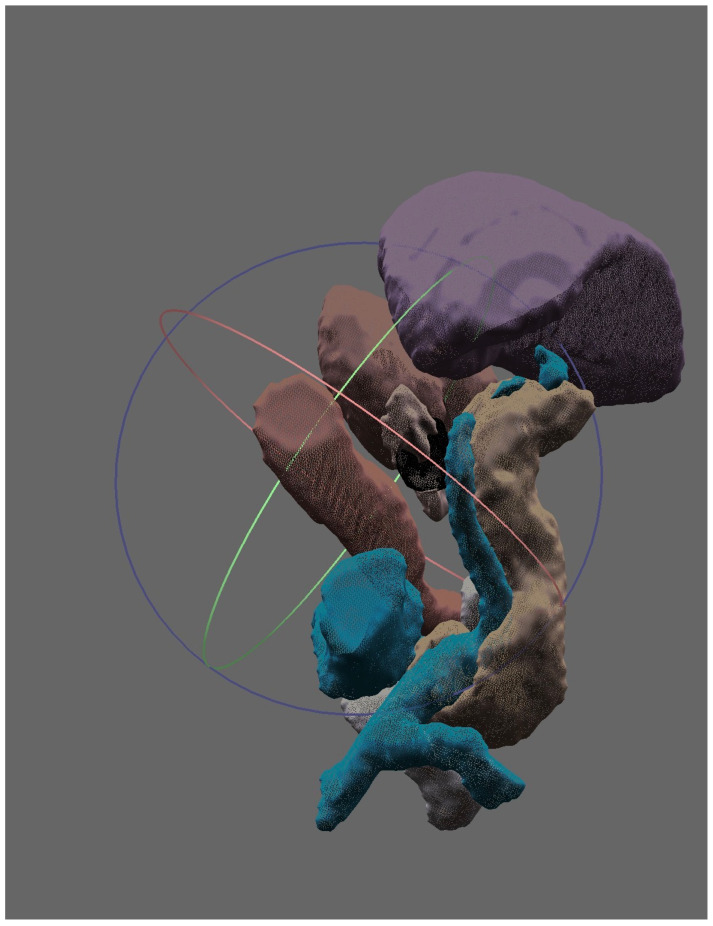
Virtual Reality image of a patient-specific 3D model reconstruction for a left-sided adrenal tumor (black).

Advances in computer-based imaging acquisition and processing have enabled accurate, patient-specific three-dimensional anatomical reconstruction for patient education, surgical training, and preoperative planning (Figure 3). These models allow the creation of fully digital environments in which clinicians can immerse themselves to simulate clinical scenarios (virtual reality, VR) or be superimposed on the real world to generate mixed visual environments or AR. This latter application has the potential to be incorporated in laparoscopic and robotic video systems.

In the past 5 years, a systematic review by Di Lorenzo et al. on 3D image applications for diagnosis and treatment of adrenal disease showed the advantages of these tools in adrenal surgery, including better tumor/normal tissue and tumor/adrenal vein relation spatial comprehension, allowing for a safer cortical-sparing adrenalectomy, a shorter operative time, and less intraoperative bleeding. Also, the application of this technology for percutaneous adrenal ablation planning has been associated with fewer needle insertions, a lower complication rate, and better local disease control, especially in large adrenal tumors or complex locations. At the end, the authors emphasize the evolving role and promising results of virtual and augmented reality in adrenal surgery, as well as the need for clinical research [78].

In the next decade, VR and AR are expected to play an expanding role in MI adrenalectomy, especially during surgical planning and training.

## 4. Discussion

This review highlights the evolution and current trends in MI adrenalectomy, reflecting how advances in technology, refined surgical techniques, and improved perioperative management have reshaped adrenal surgery over the last few decades. LA remains the gold standard for most benign and functional adrenal tumors, while alternative MI approaches—such as RPLA, LHAA, and LESSA—offer tailored solutions for specific clinical scenarios. Accumulating evidence points to considering patient and tumor characteristics, surgeon expertise, and institutional resources as the most important factors in choosing a specific MI surgical approach. On the other hand, the increasing adoption of RA, particularly in high-volume centers, underscores its potential advantages in ergonomics, dexterity, and the ability to perform complex dissections. However, the higher cost burden and borderline benefits for patients continue to limit its widespread implementation. Importantly, for specific tumors such as pheochromocytomas, emerging data suggest modest perioperative benefits of RA and RPLA, challenging long-standing dogma regarding routine alpha-blockade and highlighting the need for individualized, multidisciplinary decision-making.

As we tried to summarize, there are many options for performing minimally invasive adrenalectomy. Due to the very small number of randomized studies available, it is difficult to perform a head-to-head comparison and provide recommendations on the best surgical approach for a given patient. For instance, it is assumed that the posterior approach is faster than the lateral transabdominal approach. This assumption arose mainly from the lack of studies comparing the two approaches in similar patients and from the fact that, in most cases, smaller tumors in patients with easier anatomies have been selected for the posterior approach. When we conducted a propensity-matched comparison, we did not demonstrate any differences in perioperative outcomes between these 2 approaches [79]. A similar dilemma exists for laparoscopic versus robotic adrenalectomy. Although additional data from large-volume randomized trials are necessary, it is evident that both approaches may yield similar perioperative outcomes for straightforward adrenalectomies. However, for more complex adrenalectomies, the robotic approach seems to offer a lower risk of deviating from the original minimally invasive plan. Furthermore, for all comers, ergonomics seem better with the robotic approach.

Perioperative management of pheochromocytoma remains an area of ongoing controversy and evolving practice. Recent evidence challenges the routine use of alpha-blockade drugs, pushing for a paradigm shift in adrenal surgery for these tumors. Nevertheless, current evidence remains retrospective and heterogeneous, preventing strong conclusions on alpha-blockade omission in these patients. A more individualized approach is advised at this moment, and only under the scope of multidisciplinary expert centers.

Regarding malignant disease, particularly ACC, MI adrenalectomy may offer perioperative benefits in carefully selected early-stage tumors, but open surgery continues to represent the reference standard for locally advanced disease. An alternative minimally invasive approach in malignant cases is hand-assisted laparoscopic adrenalectomy, which could be considered.

The integration of adjunctive technologies such as LUS, ICG fluorescence, AI, VR, and AR reflects a paradigm shift toward precision adrenal surgery and is one of the most rapidly evolving areas in this surgical field. These tools aim to compensate for limitations of MI techniques by enhancing anatomical orientation, tissue discrimination, intraoperative decision-making, and surgical training. However, the evidence on these technologies remains largely limited to feasibility studies, retrospective cohorts, and proof-of-concept experiences, and their true impact on perioperative outcomes, oncological safety, and cost-effectiveness remains unclear.

On the other hand, partial adrenalectomy represents an increasingly used ancillary strategy for hereditary and bilateral pheochromocytomas, among other indications, as preservation of adrenal cortical function may substantially reduce long-term steroid dependency and adrenal crisis risk. However, evidence consistently demonstrates a higher local recurrence rate compared with total adrenalectomy. Based on this data, patient selection, genetic background, expected bilaterality of tumors, and the patient’s desire to undergo lifelong surveillance are critical factors in surgical decision-making for these cases.

## 5. Conclusions

In summary, MIS adrenalectomy has become the standard of care for the surgical treatment of most adrenal tumors. Of the large menu of MI techniques available, each and every one of them offers distinct advantages and limitations depending on patients’ characteristics, tumor or disease features, and institutional factors. Current evidence supports the safety and effectiveness of these approaches in carefully selected patients; however, the predominance of retrospective data or secondary types of studies and the limited number of randomized controlled trials continue to restrict definitive conclusions regarding the superiority of one technique over the others.

The future of adrenal surgery will rely on increasingly personalized and technology-assisted surgical strategies that will integrate advanced intraoperative imaging, fluorescence, and AI-guided virtual and augmented reality systems to improve surgical precision and decision-making. Although early reports are promising, high-quality studies remain necessary before these innovations can be broadly incorporated in everyday clinical practice.

## Figures and Tables

**Table 1:** Comparative Overview of Minimally Invasive Adrenal Surgery Techniques: Perioperative Outcomes, Advantages, Disadvantages, and Surgical Applicability.

Approach	Operative Time	Length of Stay	Complication/Conversion Rate	Advantages	Disadvantages	Best Applicability
**CLA**	**Operative time:** ~90–150 min [3,13]	**LOS:** 1–3 days [3,13]	**Complication rate:** ~5–10% [3,13] **Conversion rate:** ~1.5–2% [3,13,24,25] **Return to activity:** 2–4 weeks [3,13]	Gold-standard approach; widely reproducible [4,18] Large working space; familiar anatomy [18] Suitable for large tumors (>6 cm) and bilateral disease [3,18] Feasible after prior retroperitoneal surgery [18]	Requires visceral mobilization [18,23] Not ideal after prior abdominal surgery [18] Higher conversion rate than RPLA in some series [24,25] Longer recovery vs. RPLA in RCT data [28,29] Risk of incisional hernia at port sites [28]	First-line for most benign adrenal pathologies [4,18] Large (>6–8 cm) or complex tumors [3,18] Bilateral adrenalectomy (with repositioning) [18] When retroperitoneal approach is contraindicated [18,25] Surgeons without retroperitoneoscopic experience [18,27]
**LESSA**	**Operative time:** ~120–160 min [14,15]	**LOS:** 1–3 days [14,15]	**Complication rate:** ~5–8% [14,15] **Conversion rate:** ~3–5% [14,15] **Return to activity:** 1–3 weeks [14,15]	Superior cosmetic outcome (single scar) [14,15,16] Lower postoperative pain scores vs. CLA [14,15] Reduced analgesic consumption [14] Feasible via transperitoneal or retroperitoneal route [14,15]	Significantly longer operative time [14,15] Steep learning curve [15] Higher equipment cost; limited availability Restricted instrument triangulation in confined field Limited RCT evidence; no large-scale trials	Small to medium benign adrenal lesions (<6 cm) [14,15] Patients with high cosmetic concern [16] Experienced surgeons with dedicated single-port training [15] Not recommended for large or malignant tumors [14,15]
**RPLA**	**Operative time:** ~60–120 min [23,26]	**LOS:** 1–3 days [23,26,28,29]	**Complication rate:** ~4–8% [23,26,30] **Conversion rate:** ~1–3% [24,25,28,29] **Return to activity:** 1–2 weeks [28,29]	No visceral mobilization required [18,23] Shorter operative time vs. LTA (RCT-confirmed) [28,29] Lower postoperative pain; faster recovery [23,28,29] Bilateral adrenalectomy without repositioning [18,23] Feasible after prior abdominal surgery [18,23] Highest safety SUCRA ranking in network meta-analysis of RCTs [4]	Limited working space (challenging in BMI >30 or tumors >6–8 cm) [22,23] Steep learning curve; reverse anatomical orientation [27] Difficult for very large or locally advanced tumors [23,26] Less widespread training infrastructure [27] RCT evidence rated low certainty by Cochrane GRADE [30]	Preferred for small to medium tumors (<6–8 cm) [23,24,25] Bilateral adrenal disease [18,23] Patients with prior abdominal surgery [18,23] High-volume centers with dedicated retroperitoneoscopic training [27]
**LHAA**	**Operative time:** ~120–180 min [32,33]	**LOS:** 3–5 days [32,33]	**Complication rate:** ~8–12% (limited data; large/complex tumors) [32,33] **Conversion rate:** Low; hand access reduces open conversion risk [32,33] **Return to activity:** 2–4 weeks [32,33]	Tactile feedback facilitates dissection in complex cases [31,33] Enhanced hemostatic control via manual compression [32,33] Reduces capsular disruption risk vs. CLA for large tumors [32] Potentially shorter learning curve than conventional laparoscopy [33] Preserves MIS benefits while adding manual control [31,32,33]	Larger incision required for hand access [31,33] Repositioning may be needed for bilateral disease [31] Higher wound morbidity than other MIS approaches [33] Evidence restricted to pheochromocytoma subgroup only [32,33]	Large pheochromocytomas (>6 cm) with high hemodynamic risk [33] Malignant or suspected malignant adrenal tumors requiring oncologic control [32,33] Cases with dense adhesions or complex anatomy [32,33] Surgeons transitioning from open to MIS technique [33] Not recommended as first-line for routine benign lesions [32,33]
**RA**	**Operative time:** ~120–180 [11,12,39,41]	**LOS:** 1–3 days [11,12,39,41]	**Complication rate:** ~4–8% [11,12,37,39,41] **Conversion rate:** ~0–5% [41,47] **Return to activity:** 1–3 weeks [39,41]	Superior 3D visualization and instrument dexterity [39,41] Reduced surgeon fatigue and ergonomic strain (RCT-confirmed) [41] Favorable for right-sided tumors near IVC [39,44] Lower conversion rate vs. CLA in RCT data [41] Enables partial adrenalectomy in selected cases [43,44] Shorter learning curve vs. conventional laparoscopy for complex cases [38,39]	Substantially higher acquisition and per-case costs [39,41] Absence of haptic/tactile feedback [44] Longer docking and setup time [11,12] Limited availability in non-tertiary centers [39]	Right-sided tumors with proximity to IVC [39,44] Large, complex tumors [39,40,44] Obese patients (BMI >30) with restricted operative field [39,44] Bilateral adrenal disease [39,44] Partial adrenalectomy in hereditary syndromes (MEN2, VHL, familial pheochromocytoma) [43,44] High-volume robotic centers with trained teams [38,39]

Abbreviations: CLA, conventional laparoscopic adrenalectomy; LESSA, laparo-endoscopic single-site adrenalectomy; RPLA, retroperitoneoscopic adrenalectomy; LHAA, laparoscopic hand-assisted adrenalectomy; MIS, Minimally invasive surgery RA, robotic adrenalectomy; LOS, length of stay; IVC, inferior vena cava; MEN2, multiple endocrine neoplasia type 2; VHL, von Hippel-Lindau syndrome; BMI, body mass index; RCT, randomized controlled trial; SUCRA, surface under the cumulative ranking curve. Rehabilitation data represent pooled ranges derived from meta-analyses and large comparative series. Operative times and LOS vary with tumor size, surgeon experience, and institutional volume. All figures should be interpreted within the context of the evidence quality limitations described in Section 3.1.6.

## Data Availability

No new data were created or analyzed in this study. Data sharing is not applicable.

## Abbreviations

The following abbreviations are used in this manuscript:

ACC	Adrenocortical adenoma
AI	Artificial intelligence
AR	Augmented reality
BMI	Body mass index
CI	Confidence interval
CLA	Conventional laparoscopic adrenalectomy
ENSAT	European network for the study of adrenal tumors
ICG	Indocyanine green
IVC	Inferior vena cava
LA	Laparoscopic adrenalectomy
LESSA	Laparo-endoscopic single-site adrenalectomy
LHAA	Laparoscopic hand-assisted adrenalectomy
LND	Lymph node dissection
LOS	Length of stay
LTA	Lateral transabdominal adrenalectomy
LUS	Laparoscopic ultrasound
MEN2	Multiple endocrine neoplasia type 2
MI	Minimally invasive
ML	Machine learning
OA	Open adrenalectomy
OR	Odds ratio
PA	Partial adrenalectomy
RA	Robotic adrenalectomy
RCT	Randomized clinical trial
RPLA	Retroperitoneoscopic laparoscopic adrenalectomy
RR	Relative risk
SC	Subcostal
SILA	Single-incision laparoscopic adrenalectomy
SUCRA	Surface under the cumulative ranking curve
TU	Trans-umbilical
VHL	Von Hippel Lindau syndrome
VR	Virtual reality
WMD	Weighted mean difference
